# Comparative transcriptional analysis of flavour-biosynthetic genes of a native *Saccharomyces cerevisiae* strain fermenting in its natural must environment, vs. a commercial strain and correlation of the genes’ activities with the produced flavour compounds

**DOI:** 10.1186/s40709-019-0096-8

**Published:** 2019-08-05

**Authors:** Maria Parapouli, Afroditi Sfakianaki, Nikolaos Monokrousos, Angelos Perisynakis, Efstathios Hatziloukas

**Affiliations:** 1 Laboratory of Molecular Biology, Department of Biological Applications & Technologies, University of Ioannina, 451 10 Ioannina, Greece; 2 Laboratory of Biochemistry, Department of Chemistry, University of Ioannina, 451 10 Ioannina, Greece; 3 Department of Soil Science of Athens, Institute of Soil and Water Resources, Hellenic Agricultural Organization-DEMETER, 141 23 Athens, Greece

**Keywords:** Indigenous *Saccharomyces cerevisiae*, Higher alcohols biosynthesis, Ester biosynthesis, Gene expression, qRT-PCR

## Abstract

**Background:**

During alcoholic fermentation, *Saccharomyces cerevisiae* synthesizes more than 400 different compounds with higher alcohols, acetate esters of higher alcohols and ethyl esters of medium-chain fatty acids being the most important products of its metabolism, determining the particular flavour profile of each wine. The concentration of the metabolites produced depends to a large extent on the strain used. The use of indigenous strains as starting cultures can lead to the production of wines with excellent organoleptic characteristics and distinct local character, superior in quality when compared to their commercial counterparts. However, the relationship of these wild-type genotypes, linked to specific *terroirs*, with the biosynthetic profiles of flavour metabolites is not completely clarified and understood. To this end, qRT-PCR was employed to examine, for the first time on the transcriptional level, the performance of an indigenous *Saccharomyces cerevisiae* strain (Z622) in its natural environment (Debina grape must). The expression of genes implicated in higher alcohols and esters formation was correlated with the concentrations of these compounds in the produced wine. Furthermore, by applying the same fermentation conditions, we examined the same parameters in a commercial strain (VL1) and compared its performance with the one of strain Z622.

**Results:**

Strain Z622, exhibited lower concentrations of 2-methylbutanol, 3-methylbutanol and 2-phenyl ethanol, than VL1 correlating with the elevated expression levels of transaminase and decarboxylase genes. Furthermore, the significantly high induction of *ADH3* throughout fermentation of Z622 probably explains the larger population numbers reached by Z622 and reflects the better adaptation of the strain to its natural environment. Regarding acetate ester biosynthesis, Z622 produced higher concentrations of total acetate esters, compared with VL1, a fact that is in full agreement with the elevated expression levels of both *ATF1* and *ATF2* in strain Z622.

**Conclusions:**

This study provides evidence on the transcriptional level that indigenous yeast Z622 is better adapted to its natural environment able to produce wines with desirable characteristics, i.e. lower concentrations of higher alcohol and higher ester, verifying its potential as a valuable starter for the local wine-industry.

**Electronic supplementary material:**

The online version of this article (10.1186/s40709-019-0096-8) contains supplementary material, which is available to authorized users.

## Background

The main organism for the wine industry, widely known as “the wine-yeast”, is *Saccharomyces cerevisiae*, since predominantly strains of this species can survive the growing concentration of ethanol during fermentation [1, 2]. Consequently, it is mainly the metabolic activity of *S. cerevisiae* that determines the composition of the “flavour bouquet of fermentation-fermentative flavour”, and hence the quality of the final product [2–4].

Higher alcohols form the largest group of compounds synthesized by yeast during alcoholic fermentation [5]. Typical representatives of this group include 2-methylpropanol (isobutanol), 2-methylbutanol (amyl alcohol), 3-methylbutanol (isoamyl alcohol) and 2-phenylethanol [6]. These compounds contribute alcoholic, marzipan and rose aromas to the wine bouquet [6]. However, their effect is positive, if present at a concentration below 300 mg l^−1^; above this level the pungent odour is profound [6–8]. Higher alcohols are synthesized by *S. cerevisiae* cells via the Ehrlich pathway, which was first reported in 1907 [9] and later modified by Neubauer and Fromherz in 1911 [10]. This biosynthetic pathway consists of three steps: first, amino acids are deaminated to the corresponding α-ketoacids, in reactions catalyzed by transaminases, encoded by the genes *ARO8*, *ARO9*, *BAT1* and *BAT2* [6, 11, 12], with the latter being reported as the dominant gene for higher alcohols production [8]. In a second, decarboxylation step, α-ketoacids are converted to their corresponding aldehydes. Here, five decarboxylases are involved encoded by the genes *PDC1*, *PDC5*, *PDC6*, *ARO10* and *THI3* with the role of Thi3p being regulatory rather than catalytic [6, 11]. During the third step, alcohol dehydrogenases, such as Adh1p to Adh6p and Sfa1p catalyze the reduction of aldehydes to their corresponding higher alcohols [6, 11].

The group with the highest impact for the wine flavour, contributing with fruity aromas and determining to a great extent the distinct character of the final product is the group of esters [7]. The most important wine esters are ethyl acetate (“solvent”-like aroma), isoamyl acetate (“fruity” and “banana” aromas), ethyl caproate and ethyl caprylate (“sour apple” aroma), and 2-phenylethyl acetate (“flowery,” “roses,” and “honey” aromas) [8]. Fermentation-derived esters contributing to wine aroma form two categories: the acetate esters of higher alcohols and the ethyl esters of medium-chain fatty acids (MCFA). Acetate esters are formed intracellularly by an alcohol acetyltransferase (AATase): two such enzymes have been identified in the *S. cerevisiae* proteome, i.e. AATase I and AATase II encoded by genes *ATF1* and *ATF2*, respectively [6–8, 12]. Furthermore, *EAT1* is a recently identified gene encoding Eat1p (an ethanol acetyltransferase) [13] reported to have the potential to produce acetate and propanoate esters [14]. However, Holt et al. limited the contribution of eat1p to ethyl acetate formation [15]. With regard to acetate ester hydrolysis, the only enzyme identified in the *S. cerevisiae* proteome is isoamyl acetate-hydrolyzing esterase (Iah1p) [6–8, 12]. Most of medium chain fatty acid (MCFA) ethyl ester biosynthesis during must fermentation is catalyzed by two enzymes named Eht1p and Eeb1p [6–8, 12], both possessing an acyl-coenzymeA: ethanol *O*-acyltransferase (AEATase) activity, as well as an esterase activity [7, 8].

It is well known that the production levels of the metabolites that determine the flavour profile of the wine are variable and depend on the *S. cerevisiae* strain used [1, 4, 16–18]. As Sipiczki reported, over the last 30 years, a large number of studies have proved that *S. cerevisiae* wine-producing strains exhibit a very high degree of diversity, differing significantly in both the genotype and their oenological capacities (phenotype) [19]. The indigenous *S. cerevisiae* strains, which are representatives of the microflora of vines and local wineries, are considered as a good source for isolating strains with desirable oenological characteristics [20], able to produce wines of stylistic distinction, uniqueness and originality, characteristic of the geographical region of origin (*terroir*) [1, 21]. However, the relationship of these wild-type genotypes, linked to specific *terroirs*, with the biosynthetic profiles of flavour metabolites is not completely clarified and understood [22].

Although, in recent years, there have been numerous reports on *S. cerevisiae* transcriptomics during wine fermentation, there are still only a few comparative transcriptional studies employing different strains of *S. cerevisiae* [23–28]. Among them, the reports correlating the gene expression with the synthesis of volatile flavour compounds are even less [23, 25, 27]. Furthermore, natural grape must was used as the fermentation medium in only one of the previous studies [24]. In addition, given that the previous studies have been carried out using laboratory or industrial strains of *S. cerevisiae*, the experimental conditions generated are not representative of neither the indigenous flora nor its natural environment and therefore do not adequately reflect the expression and function of genes in the biotechnologically interesting genotypes/phenotypes of the indigenous strains.

In the present study, we have investigated the correlation of transcriptional activity of 18 genes involved in higher alcohol and ester biosynthesis, with the concentrations of the aromatic products, of an indigenous to Debina must *S. cerevisiae* strain (Z622) [29], which is regularly used by a wine producing company. Quantification of transcriptional levels was determined using quantitative real-time PCR (qRT-PCR), at three different time points of Debina must fermentations, while concentration of volatile compounds, at the same time points of fermentation, was determined using Gas Chromatography-Mass Spectroscopy (GC–MS). These data were compared with the corresponding ones, originating from the same Debina must, fermented by a commercial *S. cerevisiae* strain (VL1), which is also routinely used by local wineries for the production of Debina wines. The choice of qRT-PCR employment vs. other powerful approaches (e.g. microarray analysis, [23–26, 28]) was based on reports, in which the former method guarantees greater accuracy [30, 31] and is considered as a desirable element for the validation of microarray results [32].

To our knowledge, this is the first report of quantification by qRT-PCR of the expression of 18 genes, known to participate in wine bouquet formation, and correlation with the concentrations of flavour compounds, in a *S. cerevisiae* strain indigenous to a specific must.

## Results

### Must fermentation parameters

**Fig. 1:**
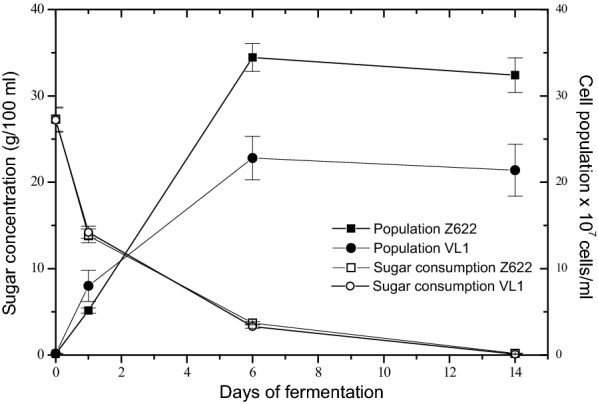
Fermentation profiles of *S. cerevisiae* strains Z622 and VL1. Statistical evaluation was performed using the program Microcal Origin 5.0 (Microcal Software, Inc.)

The wines produced by strains Z622, or VL1 possessed similar physicochemical characteristics (ethanol 11% v/v, reducing sugars 0.1–0.2 g l^−1^, density 0.990–0.991 g l^−1^, SO_2_ concentration 90–100 mg l^−1^, total acidity 5.5–5.7 g l^−1^). Furthermore, the two *S. cerevisiae* strains utilized over 99% of the initial sugar concentration within 14 days of fermentation and exhibited similar growth and ethanol production profiles (Fig. 1, Table 1), although strain Z622 reached a higher cell density (3.5 × 10^8^ cells ml^−1^) than strain VL1 (2.2 × 10^8^ cells ml^−1^) (Fig. 1). Cell growth was monitored by two different methods, qRT-PCR and plating, both proved to be of equal accuracy for cell counts, with results correlating very good with each other (R^2^ = 0.98591 for VL1 and R^2^ = 0.99985 for Z622). However, final cell counts by qRT-PCR, were obtained in a much shorter time compared with plating methodology (3 h versus 2 days, respectively).

**Table 1:** Yeast cell samples

Day of fermentation	Sugar concentration	% sugar consumption	Ethanol concentration	Cell samples V = VL1, Z = Z622	
0 (inoculation)	273 g l^−1^	0	0 g per 100 ml		
1	> 142 g l^−1^	47%	≈ 6	V1	Z1
6	> 40 g l^−1^	85%	≈ 10	V2	Z2
14	> 2 g l^−1^	99%	≈ 11	V3	Z3

### Gene expression analysis

The target genes studied for expression during Debina must fermentation by strains Z622 and VL1 are presented in Table 2. Gene expression was quantified at three different time points: (i) samples V1 and Z1 correspond to day 1 (early fermentation), sugar consumption > 47% and early exponential growth phase, (ii) samples V2 and Z2 correspond to day 6 (mid fermentation), sugar consumption > 85% and late exponential to early stationary growth phase and (iii) samples V3 and Z3 correspond to day 14 (late fermentation), sugar consumption > 99% and stationary growth phase (Fig. 1, Table 1). Amongst the six potential reference genes tested (Table 2), genes *ACT1* and *UBC6* were selected as follows: based on ANOVA statistical analysis (Additional file 1) data of day-6 expression values were normalized against *ACT1*-values, while day-14 values were normalized against *UBC6* expression values (see “Discussion”).

**Table 2:** Genes and primers used in this study

Gene	Product/function	Primer pair (forward/reverse)	Product size	Primer references
Candidate reference genes				
5.8SrRNA-ITS2	5.8S rRNA	atcgaatttttgaacgcacattg/cgcagagaaacctctctttgga	175	Hierro et al. [51]
*ACT1*	β-actin [e]/structural protein	gccttctacgtttccatcca/ggccaaatcgattctcaaaa	153	Vaudano et al. [34]
*18S*	18S rRNA	tcactacctccctgaattaggattg/agaaacggctaccacatccaa	72	Vaudano et al. [34]
*ALG9*	Mannosyltransferase activity/protein amino acid glycosylation	cacggatagtggctttggtgaacaattac/tatgattatctggcagaggaaagaacttggg	162	Teste et al. [37]
*TAF10*	RNA Pol II transcription factor activity/transcription initiation and chromatin modification	atattccaggatcaggtcttccgtagc/gtagtcttctcattctgttgatgttgttgttg	141	Teste et al. [37]
*TFC1*	RNA Pol III transcription factor activity/transcription initiation on Pol III promoter	gctggcactcatatcttatcgtttcacaatgg/gaacctgctgtcaataccgcctggag	223	Teste et al. [37]
*UBC6*	Ubiquitin-protein ligase activity/ER-associated protein catabolic process	gatacttggaatcctggctggtctgtctc/aaagggtcttctgtttcatcacctgtatttgc	272	Teste et al. [37]
Target genes				
*ATF1*	Alcohol acetyltransferase Ι/acetate ester biosynthesis	caaggtaatgtgcgatcgtg/acccaaggaaaatgcttgg	163	This study
*ATF2*	Alcohol acetyltransferase ΙI/acetate ester biosynthesis	gaggttcgcattacgcctatc/caagttgtaggacccccaga	153	This study
*EEB1*	Ethyl ester biosynthesis enzyme/ethyl ester biosynthesis/hydrolysis	accgcattacacacaggtga/agagagcgactgcagcattt	166	This study
*EHT1*	Ethanol hexanoyl transferase/ethyl ester biosynthesis/hydrolysis	gaaggatggcctcgtttaca/cactgcgagacaggttttca	163	This study
*IAH1*	Isoamyl acetate-hydrolyzing esterase/ester hydrolysis	cccttcgtggctttgaataa/ttgggatgatattgggggta	158	This study
*BAT1*	Branched chain amino acid transaminase/deamination of branched chain amino acids	catccaagccaagaccaaat/cacaagcagatgggtcaaga	147	This study
*BAT2*	Branched chain amino acid transaminase/deamination of branched chain amino acids	ctggatttaaggcggtcaga/gttggtaaccccttgaagca	141	This study
*PDC1*	Puryvate decarboxylase/decarboxylation of α-ketoacids	agctaacgctgctgtcccag/gtggtgaaaccaatggaacc	195	This study
*PDC5*	Puryvate decarboxylase/decarboxylation of α-ketoacids	ttctgaaaccactgccatga/ttcaacaacagttctaacaacttcagc	223	This study
*PDC6*	Puryvate decarboxylase/decarboxylation of α-ketoacids	caacgacggctacactatcg/ctctgaatcagtggttaaggca	169	This study
*ARO10*	Phenylpuryvate decarboxylase/decarboxylation of α-ketoacids	aaccgatcagcaacaattcc/aggccagctgattcaacact	146	This study
*THI3*	alpha-ketoisocaproate decarboxylase/decarboxylation of α-ketoacids	agaatttagcatgccgttgc/cgcctacaccaaaggttgtt	152	This study
*ADH1*	Alcohol dehydrogenase 1/reduction of aldehydes to higher alcohols	cggtgctgttctaaaggcc/tggacttgacgacttggttg	179	This study
*ADH2*	Alcohol dehydrogenase 2/reduction of aldehydes to higher alcohols	tagcgcagtcgttaaggctac/gctctgttccccacgtaaga	213	This study
*ADH3*	Alcohol dehydrogenase 3/reduction of aldehydes to higher alcohols	caacattgttcaccaggcgt/aatgcagcttccccttattc	129	This study
*ADH4*	Alcohol dehydrogenase 4/reduction of aldehydes to higher alcohols	cagctattggtctctccggta/ccttagcattgtcgtgagca	189	This study
*ADH5*	Alcohol dehydrogenase 5/reduction of aldehydes to higher alcohols	ccttcgcaagtcattcctg/atttcaattgaaatggccaatc	187	This study
*SFA1*	Class III alcohol dehydrogenase/reduction of aldehydes to higher alcohols	tatcaggctctgatccagaagg/acatttgccacactcagcag	146	This study

**Fig. 2:**
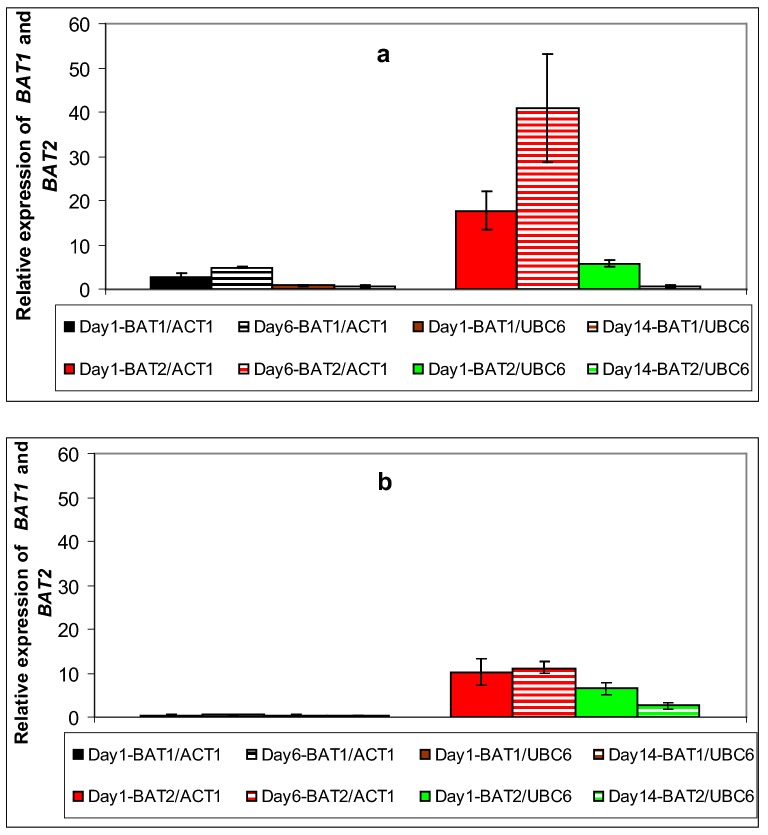
Expression of genes *BAT1* and *BAT2* during Debina must fermentations. **a** Strain VL1 and **b** strain Z622. In **a** and **b**: gene expression on day 1 was normalized against the expression levels of both *ACT1* and *UBC6* genes, whereas: (i) on day 6 expression values were normalized against *ACT1* and (ii) on day 14 against *UBC6.* In each set of values, the first bar corresponds to day 1 normalized against *ACT1*, second bar to day 6 normalized against *ACT1,* third bar to day 1 normalized against *UBC6* and fourth bar to day 14 normalized against *UBC6*

**Fig. 3:**
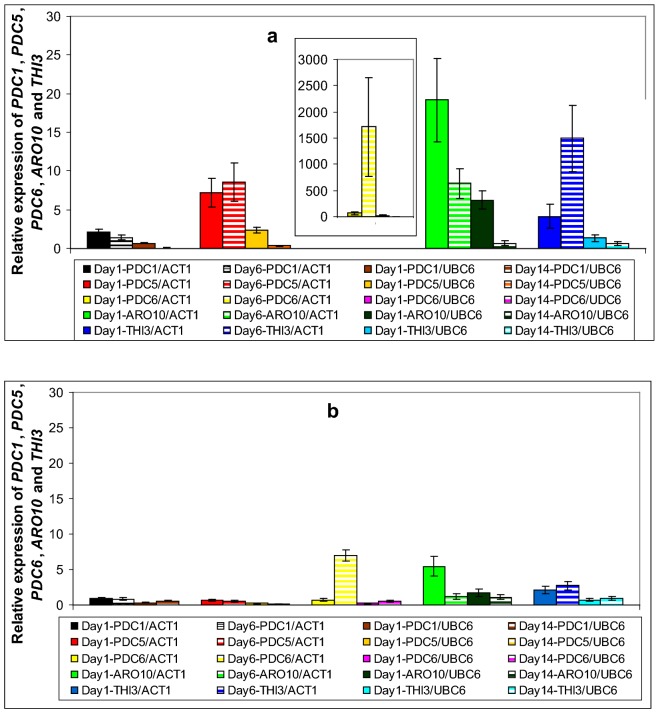
Expression of genes *PDC1, PDC5, PDC6, ARO10* and *THI3* during Debina must fermentations. **a** Strain VL1 and **b** strain Z622. In **a** and **b**: gene expression on day 1 was normalized against the expression levels of both *ACT1* and *UBC6* genes, whereas: (i) on day 6 expression values were normalized against *ACT1* and (ii) on day 14 against *UBC6.* In each set of values, the first bar corresponds to day 1 normalized against *ACT1*, second bar to day 6 normalized against *ACT1,* third bar to day 1 normalized against *UBC6* and fourth bar to day 14 normalized against *UBC6*

**Fig. 4:**
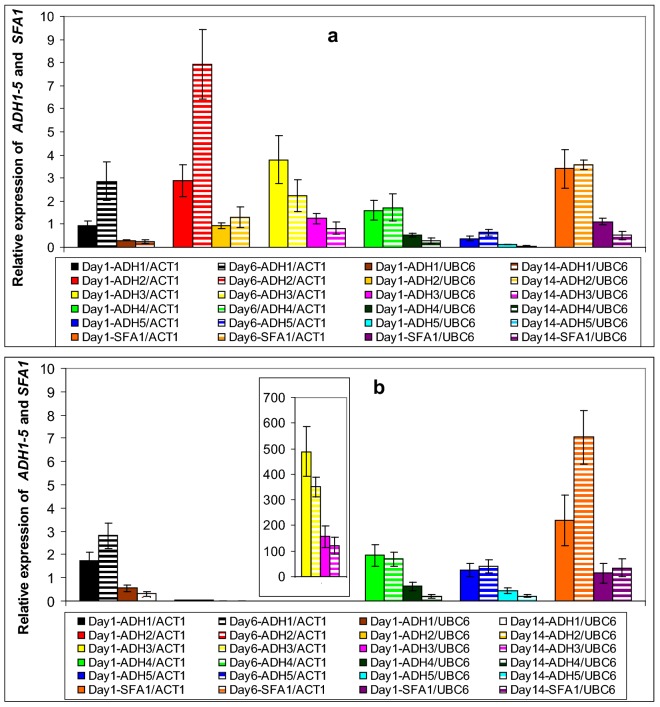
Expression of genes *ADH1*-*ADH5* and *SFA1* during Debina must fermentations. **a** Strain VL1 and **b** strain Z622. In **a** and **b**: gene expression on day 1 was normalized against the expression levels of both *ACT1* and *UBC6* genes, whereas: (i) on day 6 expression values were normalized against *ACT1* and (ii) on day 14 against *UBC6.* In each set of values, the first bar corresponds to day 1 normalized against *ACT1*, second bar to day 6 normalized against *ACT1,* third bar to day 1 normalized against *UBC6* and fourth bar to day 14 normalized against *UBC6*

**Fig. 5:**
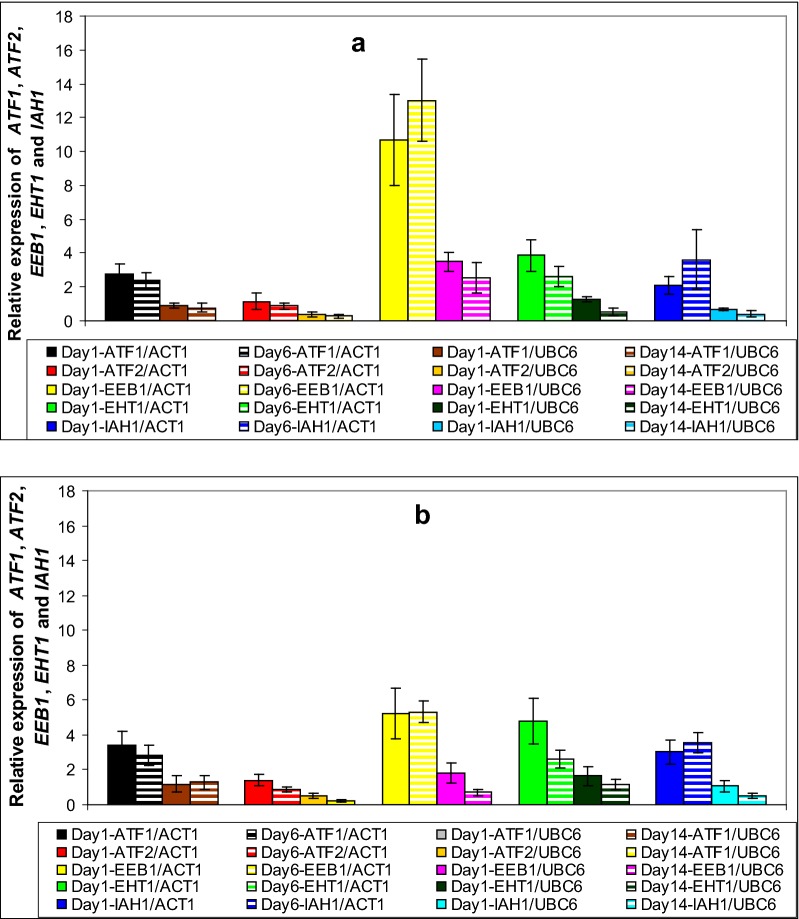
Expression of ester biosynthesis genes *ATF1, ATF2, EEB1, EHT1, IAH1* during Debina must fermentations. **a** Strain VL1 and **b** strain Z622. In (**a**) and (**b**): gene expression on day 1 was normalized against the expression levels of both *ACT1* and *UBC6* genes, whereas: (i) on day 6 expression values were normalized against *ACT1* and (ii) on day 14 against *UBC6.* In each set of values, the first bar corresponds to day 1 normalized against *ACT1*, second bar to day 6 normalized against *ACT1,* third bar to day 1 normalized against *UBC6* and fourth bar to day 14 normalized against *UBC6*

In general, the majority of the genes in this study exhibited their highest values at early and mid fermentation phases (Figs. 2, 3, 4 and 5). Specifically, *BAT1* and *BAT2* (known to participate in the first deamination step), exhibited higher induction in strain VL1, with *BAT2* being the predominantly expressed gene in both strains, throughout fermentation (Fig. 2a, b). Both genes expressed their highest values at mid fermentation: i.e. 41-fold and 11-fold induction of *BAT2* in VL1 and Z622, respectively, whereas the corresponding values for *BAT1* were V2: twofold and Z2: 1.4-fold (Fig. 2a, b). In the second step of the Ehrlich pathway, amongst all five decarboxylase-encoding genes *PDC1*, *PDC5*, *PDC6*, *THI3* and *ARO10*, the gene that yielded the highest expression value was *PDC6*, reaching an up-regulation of 1700-fold during mid fermentation in strain VLI and sevenfold in strain Z622 (Fig. 3a, b). *THI3* was the one presenting the second highest expression levels in both strains at mid fermentation (V2: 14-fold and Z2: 1.3-fold) followed by *PDC5* in VL1 (V2: 1.2-fold) (Fig. 3a, b). All other decarboxylase-encoding genes were found stable or repressed in both strains as fermentation proceeded (Fig. 3a, b). In the final stage of the Ehrlich pathway, the expression profiles indicated that the predominant enzyme throughout fermentation in strain Z622 is the product of *ADH3* with an initial 480-fold expression increase, reduced to a 120-fold at the end of fermentation (Fig. 4b). Remarkably, in the same strain almost no induction was detected throughout fermentation for *ADH2* (Fig. 4b). In strain VL1, all alcohol dehydrogenases seem to contribute to the pathway in a similar manner with genes *ADH2*, *ADH3* and *SFA1* being predominantly expressed, followed by *ADH1* and *ADH4*, whereas *ADH5* exhibited a marginal induction (Fig. 4a). Amongst them *ADH1, ADH2* and *ADH5* presented their up-regulated values at mid fermentation, *ADH4* and *SFA1* remained stable throughout exponential growth and *ADH3* exhibited reduced expression values as fermentation proceeded (Fig. 4a). Genes involved in yeast ester biosynthesis or hydrolysis include *ATF1, ATF2, EEB1, EHT1* and *IAH1* (Table 2). The expression patterns of these genes were found similar in the two studied strains (Fig. 5a, b). Regarding the genes encoding enzymes participating in ethyl ester biosynthesis in strain VL1, *EEB*1 was the one possessing the highest expression values of 10- and 13-fold at early and mid fermentation stages, respectively (Fig. 5a). In strain Z622, the corresponding values were 5.2- and 5.3-fold, respectively (Fig. 5b). On the contrary, in Z622, *EHT1* demonstrated the highest expression levels at early fermentation reaching a 4.8-fold in comparison to 3.9-fold in VL1 while at mid fermentation both strains exhibited an elevated expression of 2.6-fold (Fig. 5a, b). Furthermore, both AATases encoded by *ATF1* and *ATF*2 were found elevated in Z622, with *ATF1* exhibiting the highest expression values in both strains (Fig. 5a, b).

### Concentration profiles of volatile esters and higher alcohols in the product

The concentration of eleven compounds, the biosynthesis of which is expected to be regulated by the genes in this study, was determined at the beginning (day 1), in the middle (day 6) and at completion (day 14) of the fermentations described above. Such compounds included 4 higher alcohols (2-methylpropanol, 2-methylbutanol, 3-methylbutanol and 2-phenyl alcohol), 4 acetate esters (ethyl acetate, isoamyl acetate, hexyl acetate and phenyl acetate) and 3 ethyl esters (ethyl hexanoate, ethyl octanoate and ethyl decanoate). The concentrations of these compounds in the produced wines are summarized in Tables 3, 4 and 5. In addition, in Table 5 odour activity value (OAV) is included which is a parameter describing the potent sensory aroma contribution of the corresponding volatile compound [30]. In the beginning of fermentation, in both wines, apart from ethyl acetate, no other ester was detectable, while the higher alcohols detected did not exhibit concentrations higher than 5 mg l^−1^ (Table 3). Interestingly, in day 6 and day 14 VL1 wine products, 2-methylbutanol, 3-methylbutanol and 2-phenyl ethanol, were found to be approximately 1.1-fold to 2.1-fold higher than in Z622 wine products, which on the contrary contained higher concentrations (1.2-fold to 1.4-fold) of 2-methylpropanol (Tables 4, 5). Taking OAV into account, apparently 2-phenyl alcohol and 3-methylbutanol should have had the highest impact on the corresponding aroma in both wines (Table 5). At mid fermentation, apart from ethyl acetate, no other acetate ester was detected in both fermenting musts (Table 4). Furthermore, ethyl acetate concentrations presented a drastic decrease between mid to late fermentation (Tables 4 and 5). A closer inspection of the other acetate esters produced by each strain revealed the presence of higher levels of hexyl acetate in Z622 final wine product, while both wines contained comparable amounts of phenyl acetate and isoamyl acetate, the latter being a metabolite crucial to wine aroma according to the OAV values (Table 5). In regard to ethyl ester production, at mid fermentation stages were detected only ethyl hexanoate and ethyl octanoate, both at elevated concentrations in both wine products (Table 4). In addition, among all the metabolites studied in this article, ethyl octanoate, to which pear-like aroma is attributed, seems, according to OAV values, to have the most significant effect on wine aroma, a characteristic feature of Debina wines. Final wine fermented with strain VL1 presented the highest concentration of this ethyl ester, whereas ethyl decanoate was found in higher quantities in strain Z622-wine and ethyl hexanoate contained comparable concentrations in both wines (Table 5).

**Table 3:** Quantitative analysis of volatile compounds in the wines produced by the commercial (VL1) or the indigenous (Z622) yeast strains, on day 1

	Volatile compounds	Wine VL1	Wine Ζ622
		Compound concentration (mg l^−1^)	Compound concentration (mg l^−1^)
Alcohols detected			
1	2-methylpropanol	1.47 ± 0.07	1.67 ± 0.10
2	3-methylbutanol	1.62 ± 0.07	4.03 ± 0.24
3	2-methylbutanol	Not detected	2.78 ± 0.15
Esters detected			
4	Ethyl acetate	9.37 ± 0.66	8.73 ± 0.48

**Table 4:** Quantitative analysis of volatile compounds in the wines produced by the commercial (VL1) or the indigenous (Z622) yeast strains, on day 6

	Volatile compounds	Wine VL1	Wine Ζ622
		Compound concentration (mg l^−1^)	Compound concentration (mg l^−1^)
Alcohols detected			
1	2-methylpropanol	19.23 ± 0.96	22.67 ± 0.87
2	3-methylbutanol	116.98 ± 8.01	99.51 ± 0.34
3	2-methylbutanol	32.64 ± 0.65	25.85 ± 0.27
4	2-phenyl ethanol	46.69 ± 0.52	31.48 ± 0.33
Esters detected			
5	Ethyl acetate	119.80 ± 2.11	97.65 ± 0.34
6	Ethyl hexanoate	0.71 ± 0.34	0.44 ± 0.11
7	Ethyl octanoate	0.58 ± 0.03	0.54 ± 0.01

**Table 5:** Quantitative analysis of volatile compounds in the wines produced by the commercial (VL1) or the indigenous (Z622) yeast strains on day 14

	Volatile compounds	Wine VL1	OAV VL1	Wine Ζ622	OAV Ζ622
		Compound concentration (mg l^−1^)		Compound concentration (mg l^−1^)	
1	2-methylpropanol	9.76 ± 1.19	0.24	13.60 ± 0.17	0.34
2	3-methylbutanol	175.81 ± 0.69	5.86	121.76 ± 0.22	4.06
3	2-methylbutanol	33.29 ± 1.12	1.11	30.23 ± 0.32	1.01
4	2-phenyl ethanol	71.12 ± 1.39	7.11	33.68 ± 1.97	3.37
	Total higher oils	289.98 ± 2.00		199.26 ± 1.26	
5	Ethyl acetate	23.93 ± 1.07	3.20	32.58 ± 1.07	4.30
6	Isoamyl acetate	7.68 ± 0.15	256	7.68 ± 0.23	256
7	Hexyl acetate	–	–	0.39 ± 0.03	195
8	Phenyl acetate	1.00 ± 0.40	4.00	0.33 ± 0.47	1.32
	Total acetate esters	32.60 ± 0.82		40.98 ± 1.80	
9	Ethyl hexanoate	1.43 ± 0.45	286	2.00 ± 0.06	400
10	Ethyl octanoate	8.65 ± 0.57	4325	5.95 ± 1.46	2975
11	Ethyl decanoate	0.98 ± 0.04	4.9	1.51 ± 0.08	7.55
	Total ethyl esters	11.05 ± 0.08		9.45 ± 1.32	

*OAV* odour activity value estimated according to Molina et al. [33]

## Discussion

A very critical step before proceeding to quantitative gene expression analysis is the selection of an appropriate reference gene fulfilling the criterion of constant expression under the experimental conditions used. For this purpose, six candidate reference genes were tested (*ACT1*, *18S* rRNA gene, *ALG9*, *TAF10*, *TFC1* and *UBC6*; [33–37], as they have been reported to be constitutively expressed in yeast. qRT-PCR generated Ct values and standard curve calculations revealed that all five genes, in both strains (Z622 and VL1) presented statistically significant differences among the three stages (1, 6 and 14 days). We did not include data for reference gene *18S* rRNA, since, in preliminary experiments, we did not obtained clear results, as evaluated by melting curve analysis (data not shown). Genes *ALG9*, *TAF10* and *TFC1* showed significant statistical variation between values of the two strains and therefore their expression could not serve as normalization factor. However, *UBC6* and *ACT1* were the only ones that presented the same expression pattern in both strains (Z622 and VL1) (Additional file 1: Table S1). More specifically, ANOVA analysis showed that *UBC6* can accurately serve as a reference gene to normalize the expression values of all genes tested only for days 1 and 14 (Additional file 1: Table S1). Regarding the presentation of expression values for day 6, *UBC6* exhibited a too drastic increase, in order to serve as normalization factor for this day. On the contrary, gene *ACT1* (widely used as a reference gene [33–36], presented a much lower increase at day 6 compared to *UBC6* (e.g. for VL1, ca. 1.27 to ca. 2.08). Such a discrepancy was also previously reported [33]. To the contrary, *ACT1* expression exhibited a too drastic reduction in day 14 (Additional file 1), to be useful as a normalization factor for this day. Therefore, data of day 6 expression values were normalized against *ACT1*-values, while values of day 14 were normalized against *UBC6* expression values.

Regarding the genes involved in the first step of the Ehrlich pathway, gene *BAT2* exhibited constitutively higher expression values, than gene *BAT1* in both VL1 and Z622 (Fig. 2a, b). Our results are in agreement with published results of other authors [27, 33] both employing qRT-PCR as their analysis tool. The reverse observation has been reported based on results originating from microarray analyses [23]. These authors observed elevated expression levels of *BAT2*, when they used qRT-PCR, but not when they used a microarray analysis [23, 27]. This relative lack of accuracy of microarray transcriptional analyses has been observed also by other authors [31]. When comparing the *BAT2* expression levels in the strains in this study, VL1 exhibited higher levels than Z622 especially on day 6 (Fig. 2a, b). As reviewed by Swiegers et al. the screening of yeast strains with deletions of genes encoding decarboxylases, dehydrogenases and reductases revealed that *BAT2* is the dominant gene for higher alcohols production suggesting that the initial transamination step of the Ehrlich pathway is rate-limiting [8]. In accordance with this finding, in the second step of the pathway all decarboxylase genes in which the specificity of the pathway resides, appeared to be less active in the indigenous (Z622) yeast than the commercial (VL1) (Fig. 3a, b). Amongst the three *PDC* genes, encoding decarboxylases which have been proven to possess broad-substrate specificities with no significant differences in their enzymatic activities [38], *PDC6* was found strongly up-regulated in strain VL1 (Fig. 3a) and to a much smaller extent in Z622 (Fig. 3b). Concordant results were also reported by Beltran et al. [39] according to which, expression of *PDC6* was induced in the middle and late stages of Muscat must fermentation and correlated with enhanced higher alcohols production by a commercial strain (QA23). At the last step, in strain VL1, all five dehydrogenase encoding genes are induced, with genes *ADH2, ADH3* and *SFA1* to be most prominent (Fig. 4a). Apart from their involvement in the Ehrlich pathway, enzyme Adh1p is the primary enzyme to reduce acetaldehyde to ethanol during glucose fermentation, while Adh2p catalyzes the reverse reaction [12]. As it has also been proposed, these enzymes stabilize the NAD^+^–NADH ratio of the cell, while it has also been suggested that inter-substitution events are possible between them [40]. On the contrary, in strain Z622, *ADH2* transcriptional products were barely detectable in its cells throughout Debina must fermentation (Fig. 4b). Similar low transcript levels were also observed by Rossouw et al. [23], when they applied qRT-PCR using strains BM45 and VIN13 and by yeast strain M during wort fermentation [41].

The most striking observation was the significantly high induction of *ADH3* throughout fermentation (Fig. 4b). Adh3 is constitutively expressed during alcohol production and utilization. Its primary role is suggested to be the maintenance of redox balance, while its overexpression has been linked to cell response to various stress conditions [42, 43]. Based on these findings, the higher expression levels of *ADH3* in strain Z622 may be part of a more efficient response to the fermentation stress conditions, resulting in higher population numbers of this strain compared with the ones of VL1 (Fig. 1), and reflecting its better adaptation to its natural environment. Strain VL1, which we have previously demonstrated to produce wines with high concentrations of fusel alcohols [29], exhibited higher concentrations of 2-methylbutanol, 3-methylbutanol and 2-phenyl ethanol, in the middle and at the end of fermentation than Z622 (Tables 4, 5), correlating with the elevated expression levels of transaminase and decarboxylase genes (Figs. 2, 3). On the contrary, strain Z622 initially produced higher concentrations of fusel alcohols when compared to VL1 (Table 3), probably due to the exceptionally elevated expression of its *ADH3* gene (Fig. 4b), a unique pattern that has not been observed in previous wine fermentation studies. In addition, Z622 exhibited higher 2-methylpropanol production throughout fermentation, a fact also observed in our previous study [29]. Although, Bat2p has been proposed to play an important role in the production of the latter metabolite [35], our results do not fully confirm this proposal. Such discrepancies between studies have also been reported in the past and point to a significantly complicated relationship between the aminotransferases and the diversity of higher alcohol production in different strains [12].

Regarding acetate ester biosynthesis, Z622 produced at the end higher concentrations of total acetate esters, compared with VL1 (Table 5), a fact, that is in full agreement with the elevated expression levels of both *ATF1* and *ATF2* in strain Z622 (Fig. 5a, b). Interestingly, both strains produced high concentrations of ethyl acetate at mid fermentation, which were drastically reduced by ca. 66% in the final product (Tables 4, 5), a fact that could be due to the expression profile of *EAT1*, which was not included in this study [13, 14]. Also, remarkably, 2-methyl propyl acetate and 2-methyl butyl acetate were not detected in either wine, although their respective alcohol substrates were abundant in both strains (Table 5). This, could be explained either by the antagonistic activity of *IAH1* (esterase) [44], or due to the existence of other—as yet unknown—enzymes involved in acetate esters biosynthesis/hydrolysis, as proposed by other research groups ([14, 45] and references therein). With regard to MCFA ethyl esters levels, strain VL1 exhibited a higher ethyl octanoate production, than Z622 (Tables 4, 5), Z622 synthesized higher amounts of ethyl decanoate (Table 5), whilst both strains produced elevated concentrations of ethyl hexanoate (Tables 4, 5). In strain VL1, *EEB1* was found constitutively up-regulated in comparison to *EHT1* (Fig. 5a). In strain Z622 *EEB1* and *EHT1* possessed similar expression values on day 1 while on days 6 and 14, they were expressed in the opposite way (Fig. 5b). As it was proposed by Lilly et al. [46], overexpression of *EHT1* resulted in increased concentrations of ethyl hexanoate, ethyl octanoate and ethyl decanoate, whereas Saerens et al. [47] did not report such a correlation. Nevertheless, the latter study has demonstrated that deletion of *EEB*1 led to an 80% decrease in the concentration of ethyl hexanoate and to a 50% decrease in the concentrations of ethyl octanoate and ethyl decanoate. The fact that such enzymes possess both biosynthetic and hydrolytic capabilities [47, 48] in addition to the existence of other and unknown esterases in the *S. cerevisiae* proteome [45] does not enable us to identify in its full extent the relationship between the gene expression of *EEB*1 and *EHT*1 and MCFA ethyl esters biosynthesis. From a methodological point of view, this study was carried out in conditions very close to traditional wine-making: i.e. an indigenous strain fermenting its natural grape must. Thus, these conditions are significantly different from those used in previous studies, which investigated expression profiles of solely laboratory or industrial strains of *S. cerevisiae* fermenting mostly in synthetic media. Although the transcriptional analysis of wine fermentation was done in a single vintage season, a strong indication that this work provides reliable results, is that the flavour profile of the wines produced is identical to the corresponding profiles of wines produced in other vintages, presented in our previous study [29].

## Conclusions

To conclude, this comparative study of a wild-type and an industrial *S. cerevisiae* genotype, which presents for the first time transcriptional data of an indigenous strain (Z622), grown in its natural environment (Debina grape must), revealed differential gene expression between the two strains which is reflected on the flavour content of the produced wines. Thus, this study provides evidence on the transcriptional level that indigenous yeast Z622 is better adapted to its natural environment able to produce wines with desirable characteristics, i.e. lower fusel alcohol and higher ester concentrations [1], that render it a valuable starter for the local wine-industry. Additional studies including other wild-type yeast and musts of different origin are required to further clarify the relationship of these wild-type genotypes, with the biosynthetic profiles of flavour metabolites.

## Methods

### Must fermentations and Sampling

Filtered (0.22 μm, Corning Incorporated-Corning, USA) Debina grape must (Baumė 11.32) was fermented using two strains of *S. cerevisiae*: Z622 (indigenous to the area of Zitsa, Epirus) [29], or VL1 (commercial), each added at an initial inoculum of 10^6^ cells ml^−1^. Yeast cells from YM pre-cultures [49] were centrifuged and washed with Debina grape must prior to final inoculation, to avoid transfer of nutrients from YM to the fermentation medium. Fermentations took place in a 10 l Bioflo 110 bioreactor equipped with a cooling system (New Brunswick Scientific, New Jersey, USA), without aeration, with temperature adjusted to 18 °C. Yeast growth was monitored employing qRT-PCR technology as well as plating (cfu counting). Sugar utilization was determined by the Nelson method [50]. Triplicate samples of yeast cells were collected from each of the three different time points during fermentations, as shown in Table 1.

### DNA extraction

Yeast cells were washed with sterile water, resuspended in 1 ml of Lysis Solution (MasterPure Yeast DNA Purification kit, Epicentre Biotechnologies, Madison, USA) and disrupted in a mini Bead Beater (Biospec Products, Bartlesville, USA; 5 × 1 min vibrations, using glass beads 0.5 mm diameter). Homogenates were centrifuged at 3000 g for 5 min and supernatants containing DNA were further purified using the MasterPure Yeast DNA Purification kit (Epicentre Biotechnologies) and its quality assessed spectrophotometrically and electrophoretically. DNA was immediately diluted 1:1000 and was further used as the template in qRT-PCR reactions.

### RNA extraction and cDNA synthesis

Total RNA was extracted using the MasterPure Yeast RNA Purification kit (Epicentre Biotechnologies, Madison, USA). RNA quality and concentration were assessed spectrophotometrically and by electrophoresis in 1.2% agarose gels. For the reverse transcription reaction, cDNA was synthesized using 0.2 μg total RNA as template and the PrimeScript 1st strand cDNA synthesis kit (Takara Bio Inc, Otsu, Japan) according to the manufacturer’s recommendations. cDNA, diluted 1:10, was used as DNA template in qRT-PCR experiments, as described below.

### Primer design

To monitor the increase of the yeast population, primers for selective amplification of *S. cerevisiae* DNA (CESP-F/SCER-R) were according to Hierro et al. [51]. For gene expression analysis, primers used for the reference genes *ACT1* and *18S* were according to Vaudano et al. [34], whereas for *ALG9*, *TAF10*, *TFC1* and *UBC6* according to Teste et al. [37]. Primers for the genes *ATF1*, *ATF2*, *EEB1*, *EHT1*, *IAH1*, *BAT1*, *BAT2*, *PDC1*, *PDC5*, *PDC6*, *ARO10*, *THI3*, *ADH1*-*ADH5* and *SFA1* were designed using the Primer 3 software [52] and sequences of corresponding genes located in the GenBank database. Several sequence alignments were performed using the ClustalW multiple sequence alignment software [53], to ensure primer selectivity in the cases of *PDC1*, *PDC5*, *PDC6* and *ADH1*-*ADH5*, which exhibit a high degree of sequence identity. The primers used in this study and the size of their amplification products are shown in Table 2.

### qRT-PCR

qRT-PCR reactions using CESP-F/SCER-R primers were performed according to Hierro et al. [51]. Reactions using all other primer sets were carried out in a total volume of 20 μl that contained 2 μl of cDNA, 0.2 μM of forward and reverse primers, and 10 μl of 2× SYBR Green master mix (Takara Bio Inc, Otsu, Japan). Amplifications were performed in an Opticon2 thermocycler (MJ Research, Waltham, USA) under the following conditions: 95 °C for 3 min; 40 cycles of 95 °C for 10 s, 58 °C for 20 s, and 72 °C for 30 s; and a final extension at 72 °C for 1 min. At the end of the amplification cycle, a melting analysis was conducted to verify the specificity of the reaction. This was carried out by heating the amplification products from 50 °C to 90 °C at 0.2 °C per 0.02 s.

### Cell counting

First by RT-PCR, DNA obtained from *S. cerevisiae* cultures with a concentration of 10^6^ cells ml^−1^ was serially diluted tenfold and was used to construct a standard curve as described by Hierro et al. [51]. Second, cell counting by plating (cfu) was performed on non-selective YM plates [46]. Minimal medium agar plates (MM) [54] with lysine (as sole carbon source), which cannot support growth of *S. cerevisiae* cells ([51] and references therein), served as negative controls.

### Gene expression analysis

Expression of target genes was quantified by the standard curve method, according to the ABI PRISM Bulletin [55]. Standard curves were constructed using serial dilutions of cDNA from RNA extract of yeast cells grown in YM. The R^2^ values of all standard curves were higher than 0.98. Six genes were selected and examined as potential reference genes including *ACT1*, *18S*, *ALG9, TAF10*, *TFC1* and *UBC6*. Each sample was analyzed in triplicate.

### Wine analyses

Determination of the physicochemical parameters of the fermented wines was performed according to the official analytical methods of the European Community [56]; sample preparation was performed by diethyl ether extraction according to Lilly et al. [57]; GC–MS analysis was performed as described previously, in Parapouli et al. [29].

### Statistical analysis

In order to identify differences in the expression of the reference genes (five in total) from both strains at the three different stages of fermentation, the mean values of triplicate sample repetition were subjected to statistical analysis using one-way analysis of variance (ANOVA). In the case of significant differences in ANOVA (*p *< 0.05), post hoc comparisons by a Fischer test were further applied. When the assumptions of the ANOVA (normal distribution, independence between means, variances) were not met, data were transformed properly. All analyses were conducted using the SPSS 16 software (SPSS Inc. Released 2007. SPSS for Windows, Version 16.0, Chicago, USA).

## Additional file

**Additional file 1.** Mean values (± standard deviation) of expression of five genes from both strains selected to serve as potential reference genes and differences between the three different stages as revealed by ANOVA.

## Data Availability

All data generated or analyzed during this study are included in this published article (and its additional files)
