# Supplementary material for: Comparative transcriptional analysis of flavour-biosynthetic genes of a native Saccharomyces cerevisiae strain fermenting in its natural must environment, vs. a commercial strain and correlation of the genes’ activities with the produced flavour compounds
Source: J Biol Res (Thessalon). 2019 Aug 5;26:5. doi: 10.1186/s40709-019-0096-8 (PMC6683356; doi:10.1186/s40709-019-0096-8)
Supplement: Supplementary file 1 — Additional file 1. Mean values (± standard deviation) of expression of five genes from both strains selected to serve as potential reference genes and differences between the three different stages as revealed by ANOVA. [file 40709_2019_96_MOESM1_ESM.doc]

**Additional file 1**

Comparative transcriptional analysis of flavour-biosynthetic genes of a native *Saccharomyces cerevisiae* strain fermenting in its natural must environment, vs. a commercial strain and correlation of the genes’ activities with the produced flavour compounds.

Maria Parapouli, Afroditi Sfakianaki, Nikolaos Monokrousos, Angelos Perisynakis, Efstathios Hatziloukas

Table S1

Mean values (± Standard Deviation) of expression of five genes from both strains selected to serve as potential reference genes and differences between the three different stages as revealed by One-way ANOVA

|  | ***ALG*9** | ***TAF10*** | ***TFC1*** | ***UBC6*** | ***ACT1*** |
| --- | --- | --- | --- | --- | --- |
| Ζ1 | 1.98±0.15 **a** | 2.47±0.06 **a** | 2.14±0.41 **a** | 1.99±0.53 **a** | 0.64±0.13 **a** |
| Ζ2 | 5.52±0.97 **b** | 11.80±3.87 **b** | 11.21±0.74 **b** | 9.42±0.79 **b** | 1.40±0.15 **b** |
| Ζ3 | 1.34±0.30 **a** | 1.90±0.21 **a** | 1.36±0.57 **a** | 1.22±0.32 **a** | 0.1±0.02 **c** |
| LSD p<0.05 | *** | ** | *** | *** | *** |

|  | ***ALG*9** | ***TAF10*** | ***TFC1*** | ***UBC6*** | ***ACT1*** |
| --- | --- | --- | --- | --- | --- |
| V1 | 2.38±0.77 **a** | 2.47±0.39 **a** | 2.81±0.54 **a** | 3.25±0.23 **a** | 1.06±0.23 **a** |
| V2 | 11.86±1.76 **b** | 8.97±0.27 **b** | 10.84±0.78 **b** | 9.41±0.89 **b** | 2.00±0.36 **b** |
| V3 | 5.21±1.20 **c** | 9.13±2.63 **b** | 5.14±0.91 **c** | 5.08±1.64 **a** | 0.58±0.10 **c** |
| LSD p<0.05 | *** | ** | *** | ** | *** |

(***p<0.001; **p<0.01). The letters a, b and c correspond to statistically significant differences among the values of the two strains, e.g. all values designated a present no statistically significant difference.
